# Detrimental effects of PCSK9 loss-of-function in the pediatric host response to sepsis are mediated through independent influence on Angiopoietin-1

**DOI:** 10.21203/rs.3.rs-2521836/v1

**Published:** 2023-02-03

**Authors:** Mihir R. Atreya, Natalie Z. Cvijanovich, Julie C. Fitzgerald, Scott L. Weiss, Michael T. Bigham, Parag N. Jain, Adam J. Schwarz, Riad Lutfi, Jeffrey Nowak, Geoffrey L. Allen, Neal J. Thomas, Jocelyn R. Grunwell, Torrey Baines, Michael Quasney, Bereketeab Haileselassie, Matthew N. Alder, Patrick Lahni, Scarlett Ripberger, Adesuwa Ekunwe, Kyle R. Campbell, Keith R. Walley, Stephen W. Standage

**Affiliations:** Cincinnati Children’s Hospital Medical Center; UCSF Benioff Children’s Hospital Oakland; Children’s Hospital of Philadelphia; Children’s Hospital of Philadelphia; Akron Children’s Hospital; Texas Children’s Hospital, Baylor College of Medicine; Children’s Hospital of Orange County; Riley Hospital for Children; Children’s Hospital and Clinics of Minnesota; Children’s Mercy Hospital; Penn State Hershey Children’s Hospital; Children’s Healthcare of Atlanta at Egleston; University of Florida Health Shands Children’s Hospital; CS Mott Children’s Hospital at the University of Michigan; Lucile Packard Children’s Hospital Stanford; Cincinnati Children’s Hospital Medical Center; Cincinnati Children’s Hospital Medical Center; Cincinnati Children’s Hospital Medical Center; Cincinnati Children’s Hospital Medical Center; University of British Columbia; University of British Columbia; Cincinnati Children’s Hospital Medical Center

**Keywords:** Sepsis, Septic shock, Multiple Organ Dysfunction Syndrome, PCSK9, LDLR, Genotype, Lipoproteins, Endothelium, Endothelial dysfunction, Biomarkers

## Abstract

**Background:**

Sepsis is associated with significant mortality, yet there are no efficacious therapies beyond antibiotics and supportive care. In adult sepsis studies, PCSK9 loss-of-function (LOF) and inhibition has shown therapeutic promise, likely through enhanced low-density lipoprotein receptor (LDLR) mediated endotoxin clearance. In contrast, we previously demonstrated higher mortality in septic juvenile hosts with *PCSK9* LOF. In addition to direct influence on serum lipoprotein levels, PCSK9 likely exerts pleiotropic effects on vascular endothelium. Both mechanisms may influence sepsis outcomes. We sought to test the influence of *PCSK9* LOF genotype on endothelial dysfunction in pediatric sepsis.

**Methods:**

Secondary analyses of a prospective observational cohort of pediatric septic shock. Single nucleotide polymorphisms of *PCSK9* and *LDLR* genes were assessed. Serum PCSK9, lipoprotein, and endothelial marker concentrations were measured. Multivariable linear regression tested the influence of *PCSK9* LOF genotype on endothelial markers, adjusted for age, complicated course, and low- and high-density lipoproteins (LDL and HDL). Causal mediation analyses assessed impact of select endothelial markers on the association between *PCSK9* LOF genotype and mortality. Juvenile *Pcsk9* null and wildtype mice were subject to cecal slurry sepsis and endothelial markers were quantified.

**Results:**

474 patients were included. *PCSK9* LOF was associated with several markers of endothelial dysfunction, with strengthening of associations after exclusion of patients homozygous for the rs688 *LDLR* variant that renders it insensitive to PCSK9. Serum PCSK9 levels did not correlate with endothelial dysfunction. *PCSK9*LOF significantly influenced concentrations of Angiopoietin-1 (Angpt-1) and Vascular Cell Adhesion Molecule-1 (VCAM-1). However, upon adjusting for LDL and HDL, *PCSK9*LOF remained significantly associated with low Angpt-1 alone. Causal Mediation Analysis demonstrated that the effect of *PCSK9* LOF on mortality was partially mediated by Angpt-1 (p=0.0008). Murine data corroborated these results with lower Angpt-1 and higher soluble thrombomodulin among knockout mice with sepsis relative to the wildtype.

**Conclusions:**

*PCSK9* LOF independently influences serum Angpt-1 levels in pediatric septic shock. Angpt-1 likely contributes mechanistically to the effect of *PCSK9* LOF on mortality in juvenile hosts. Mechanistic studies on the role of PCSK9-LDLR pathway on vascular homeostasis may lead to the development of novel pediatric-specific sepsis therapies.

## Introduction

Sepsis is a major pediatric health problem resulting from a dysfunctional host response to an infection, which can further drive multiple organ dysfunctions and death. Recent studies suggest that up to 40% of global sepsis cases occurred under the age of 5, with more than 20 million cases reported worldwide in 2017.^1^ Moreover, the World Health Organization first global report on sepsis estimate that it accounts for 20% of all deaths and is the leading cause of under-5 mortality.^2^ Further, the economic burden of sepsis is staggering, with more than $7 billion spent on pediatric cases in the U.S alone.^3^ Despite this burden of disease, sepsis care remains limited to early antibiotics and organ support, with no efficacious biological therapies available.

Within the previous decade, Proprotein Convertase Subtilisin/Kexin type 9 (PCSK9) has been recognized to play a critical role in sepsis pathobiology.^4,5^ *PCSK9* loss-of-function (LOF) or pharmacologic inhibition has been demonstrated to result in increased hepatocyte low-density lipoprotein receptor (LDLR) mediated bacterial and endotoxin clearance.^6,7^ Based on these data, ongoing clinical trials will test the efficacy of commercially available PCSK9 inhibitors as novel sepsis therapeutics (NCT03869073 and NCT03634293). More recent observational data among adults and children, however, have shown contradictory results, with both *PCSK9* LOF genotype^8,9^ and very low serum PCSK9 concentrations^9–11^ being associated with equivocal or worse septic shock outcomes. Thus, it is likely that the biology of the PCSK9-LDLR pathway among critically ill patients remains incompletely understood.

Endothelial dysfunction is a key putative mechanism of organ failure in critical illness including septic shock.^12^ PCSK9 was recently shown to have pleiotropic effects on endothelial inflammation,^13,14^ in addition to its impact on the bleeding and coagulation cascades.^13,14^ It remains unknown whether these are a direct effect or are mediated through their influence on circulating lipoprotein profiles, which are also known to modulate endothelial function.^15^ A major limitation, however, is that much of the extant literature on the influence of PCSK9 on the endothelium has focused on patients and disease models of dyslipidemia. On the contrary, critical illness is associated with drastic shifts in serum lipoprotein profiles, with very low rather than high concentrations common among adults and children.^16,17^

Accordingly, given its respective contribution to sepsis pathobiology and the potential for interaction during systemic inflammation among critically ill patients, we sought to test 1) whether *PCSK9* LOF genotype was independently associated with markers of endothelial dysfunction after accounting for serum lipoprotein concentrations and 2) whether these effects had a causal impact on mortality outcomes in a large pediatric cohort of septic shock. Lastly, we sought to corroborate the association between *PCSK9* LOF genotype and endothelial markers in a juvenile murine model of sepsis.

## Methods

### Study design and patient selection:

The study protocol was approved by Institutional Review Boards of participating institutions.^19,20^ Briefly, patients under the age of 18 years were recruited from multiple pediatric ICUs (PICU) across the U.S. between 2003 and 2019. There were no study related interventions except for blood draws. Clinical and laboratory data were available between day 1 through 7. Inclusion criteria were 1) patients meeting pediatric-specific consensus criteria for septic shock,^18^ 2) available data on existing PCSK9-LDLR single nucleotide polymorphisms.^9^ Patients with both LOF and gain-of-function (GOF) mutations (n=20) and missing endothelial marker data (n=29) were excluded.

### Genotyping:

Polymerase chain reaction (PCR) experiments using TaqMan assays were performed, as previously described.^9^ Briefly, we tested for the most common *PCSK9* missense loss-of-function (LOF) variants: rs11591147 (R46L), rs11583680 (A53V), and rs562556 (V474I), the most common gain-of-function (GOF) variant rs505151 (G670E), and a variant of the LDLR gene (rs688) that renders it insensitive to changes in PCSK9.

### Serum PCSK9 concentrations:

PCSK9 concentrations were measured in serum samples collected within 24 hours of admission to the PICU (day 1) of septic shock by ELISA (R&D Systems, USA, DPC900) according to the manufacturers’ specifications, as previously detailed.

### Serum lipoprotein concentrations:

Lipoprotein profiles were measured in day 1 serum samples on a Randox RX Daytona clinical analyzer by direct clearance.

### Serum endothelial marker concentrations:

Concentrations of Angiopoietin-1 (Angpt-1), Angiopoietin-2 (Angpt-2), Tyrosine kinase with immunoglobulin-like loops and epidermal growth factor homology domains-2 (Tie-2), Intercellular adhesion molecule-1 (ICAM-1), Vascular cell adhesion molecule-1 (VCAM-1), soluble Thrombomodulin (sTM), were measured in day 1 serum by Luminex assays (R&D Systems, MN), as previously published.^19^

### Juvenile murine model of sepsis:

Our animal studies complied with the Guide for the Care and Use of Laboratory Animals published by the U.S. National Institutes of Health,^20^ and were approved by the Institutional Animals Care and Use Committee (IACUC). Established colonies of constitutive Pcsk9 null mice with C57BL/6 genetic background (*Jackson Laboratory, Pcsk9*−/−; *B6;129S6-Pcsk9tm1Jdh/J*)) and wildtype mice (*C57BL/6*) were utilized. Mice were maintained with standard housing, food, and day/night regulation. Juvenile (14-day-old) mixed sex mice were used for experiments. Cecal slurry (0.8 mg/gram body weight prepared in D5W solution) was administered via intraperitoneal (I.P.) injection with a 27-gauge needle. Sham animals received I.P. injections with equal volume of D5W. Animals received neither antibiotics nor fluid resuscitation, and were housed with dams. For sample collection, all animals were anesthetized, followed by cervical dislocation, and blood drawn by terminal cardiac puncture 16 hours after cecal slurry or sham injections — a time point before early sepsis deaths occurred in prior survival studies. Serum was stored at −80°C for molecular assays.

### Serum endothelial markers in murine studies:

Concentrations of Angpt-1 (Novus Biological), Angpt-2, and Tie-2 (R&D Systems) were determined by ELISA according to manufacturers’ instructions. Serum sTM, ICAM-1, VCAM-1, were measured by custom Luminex multiplex assay (R&D systems). Because of the limited availability of serum from juvenile animals, serum Angpt-1, Angpt-2, and Tie-2 were measured in different sets of animals. Thus, we could not estimate Angpt-2/Angpt-1 and Angpt-2/Tie-2 ratios in mice. Similarly, we did not have sufficient serum to measure lipoprotein concentrations to test their effect as mediators.

### Statistical analyses:

Statistical analyses were performed using R software (version 4.2.2).

Demographic and clinical data were summarized with percentages or median with outer limits for interquartile ranges (Q1 and Q3). Differences between groups were determined by the χ2 test for categorical variables and Kruskal Wallis non-parametric test for continuous variables. Relationship between endothelial dysfunction markers and serum PCSK9 concentrations was determined by simple linear regression. Age-related changes and a higher burden of death and multiple organ dysfunctions may potentially influence the association between patient genotype and endothelial dysfunction markers. Accordingly, multi-variable linear regression models were developed to test the influence of age, complicated course, *PCSK9* LOF genotype on endothelial markers among patients. In addition, we adjusted for low and high-density lipoprotein (LDL and HDL) concentrations in separate models.

### Causal Mediation Analyses:

To assess the causal impact of PCSK9 LOF on mortality via either its canonical effect on LDL cholesterol or via novel endothelial pathways, as marked by Angpt-1 and VCAM-1, we used Causal Mediation Analysis (R package Mediate v4.5.0).^21^ Effect sizes were reported as the average causal mediation effects (ACME), average direct effects (ADE), and the total effect which is the sum of ACME and ADE. To estimate parameters, bootstrapping with 5000 simulations was used. Significance was declared when two-sided P-values for ACME were ≤ 0.05.

### Murine endothelial markers:

Two-way ANOVA was used to test the influence of genotype (Pcsk9 null vs wildtype) and condition (sepsis vs sham) with post-hoc pairwise contrasts corrected for multiple comparisons using the Tukey HSD method. P-value of < 0.05 was considered statistically significant.

## Results

A total of 474 patients were included in this study. One hundred and ninety-five patients carried at least one *PCSK9* LOF variant. The remaining 279 patients carried either GOF variants or neither LOF nor GOF variants and served as the reference group. Table 1 shows demographic and clinical characteristics comparing patients with *PCSK9* LOF variants relative to those without. A significantly higher proportion of patients who self-identified as having Caucasian ancestry carried LOF variants. There were no differences in baseline illness severity nor co-morbidities between groups. As previously detailed,^9^ those with *PCSK9* LOF variants had significantly higher rates of complicated course, 28-day mortality, and burden of organ failures.

**Figure 1:**
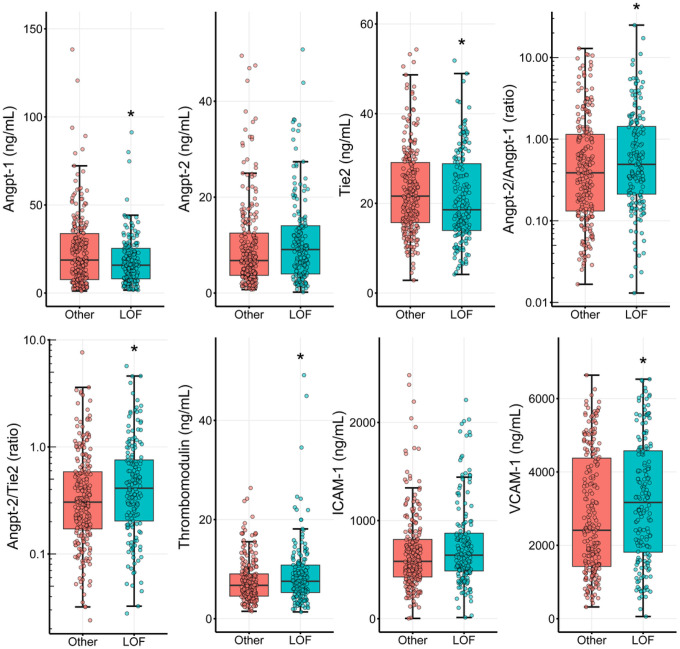
Box and whisker plots of median concentrations of serum markers of endothelial dysfunction among pediatric septic shock patient with PCSK9 loss-of-function variants relative to those without. Associations shown after exclusion of patients homozygous for rs688 LDLR variant, which renders it insensitive to PCSK9.

Figure 1 shows the association between *PCSK9* LOF genotype and markers of endothelial dysfunction tested after exclusion of patients homozygous for the rs688 *LDLR* variant, which renders the LDLR insensitive to PCSK9 signaling. Concentrations of Angpt-1 and Tie-2 were lower, while VCAM-1, sTM, and ratios of Angpt-2/Angpt-1 and Angpt-2/Tie-2 were higher, among those with *PCSK9* LOF genotype relative to those without. These data are summarized in tabular format in **Additional File 1**. Results of multivariate regression analyses to test the influence of *PCSK9* LOF genotype on markers of endothelial dysfunction are presented in Table 2. Correcting for patient age and complicated course and excluding patients with the rs688 LDLR variant, the *PCSK9* LOF genotype significantly influenced only Angpt-1 and VCAM-1 levels. sTM showed only a trend toward association with the LOF genotype. Serum PCSK9 concentrations, however, did not correlated with any endothelial dysfunction marker as shown in **Additional File 2**.

**Figure 2:**
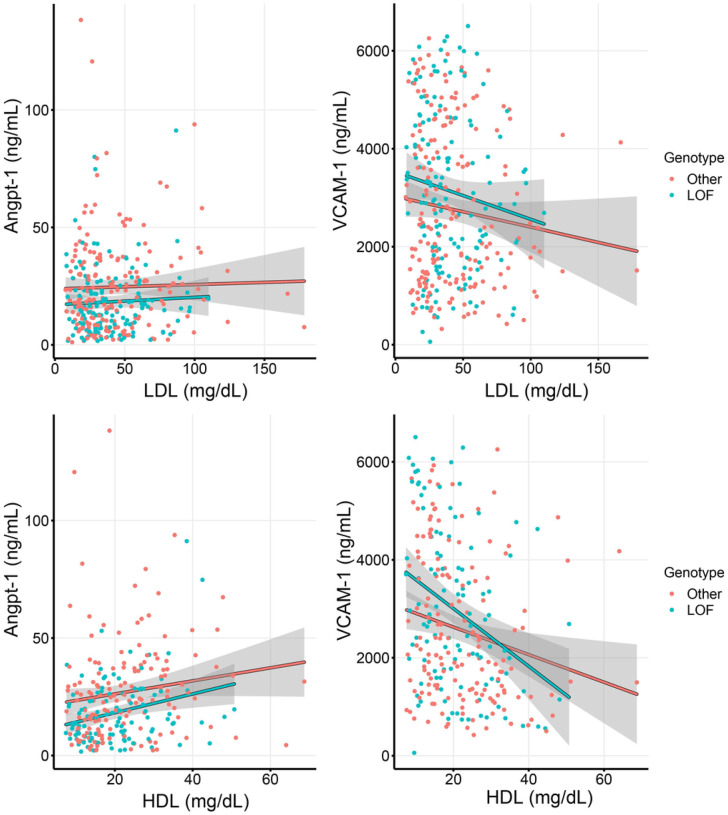
Association between PCSK9 LOF genotype, serum Angpt-1 (left panels) and VCAM-1 (right panels), across the range of serum LDL (top panels) and HDL (bottom panels) concentrations. Patients with PCSK9 LOF genotype had lower Angpt-1 irrespective of LDL and HDL, but higher VCAM-1 only at extremely low LDL and HDL concentrations.

A total of 326 patients had available data on serum LDL and HDL concentrations in addition to genotyping and endothelial marker data. The multivariate models testing the influence of serum LDL and HDL concentrations on the association between *PCSK9* LOF genotype and endothelial dysfunction markers are shown in Table 3 and **Additional File 3** respectively. Both serum LDL and HDL were independently associated with several endothelial dysfunction markers. However, after adjusting for age, complicated course, HDL, and LDL in separate models, only Angpt-1 was significantly associated with *PCSK9* LOF. Figure 2 shows the association between *PCSK9* LOF genotype, concentrations of Angpt-1 and VCAM-1, across the range of serum LDL and HDL. Angpt-1 levels were consistently lower among patients with LOF genotype irrespective of lipoprotein concentrations. However, VCAM-1 levels increased among patients with LOF genotype only at low lipoprotein concentrations.

We used Causal Mediation Analysis to determine whether the relationship between *PCSK9* LOF and previously published association with increased mortality in this cohort^9^ was a result of the known effects of PCSK9 on serum LDL concentrations or whether it was mediated by a novel endothelial pathway involving Angpt-1 or VCAM-1. We found that in each analysis the direct relationship between *PCSK9* LOF and increased mortality persisted as shown in Table 4. Angpt-1 was found to be a significant mediating variable, contributing over 12% of the effect of *PCSK9* LOF on mortality (p = 0.0008). In contrast, neither VCAM-1 nor, surprisingly, LDL levels contribute significantly to the effect of *PCSK9* LOF on mortality (p=0.17 and 0.94 respectively).

**Figure 3:**
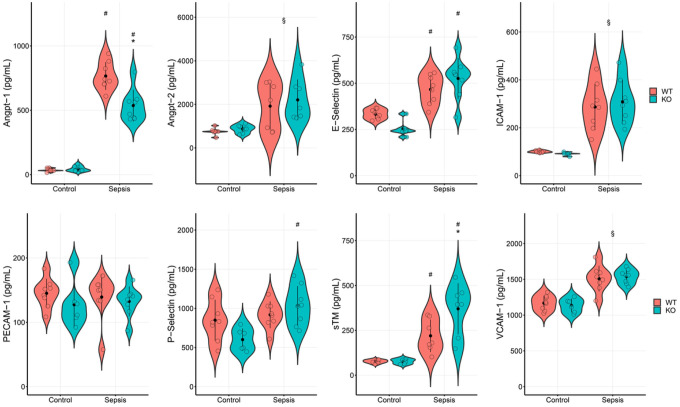
Violin plots showing results of two-way ANOVA of endothelial markers in juvenile mice by genotype (Pcsk9 null or knockout (KO, teal color) vs. wildtype (WT, rust color)) and condition (sepsis vs. control). * KO cohort statistically different from WT cohort in condition (interaction effect). # Sepsis cohort different from Control for genotype (interaction effect). † KO genotype differ from WT genotype across conditions (main effect: genotype). § Sepsis condition differs from Control across genotypes (main effect: condition).

Figure 3 shows concentrations of endothelial dysfunction markers among experimental groups in juvenile murine sepsis studies. Unsurprisingly, septic animals had higher endothelial dysfunction relative to sham animals. However, genotype specific differences in endothelial markers among septic animals were observed only for Angpt-1 and sTM, with lower and higher levels respectively noted among *Pcsk9* null mice relative to the wildtype

## Discussion

In this study, we build upon our previous observations that *PCSK9* LOF genotype among children with septic shock and genetic ablation in juvenile mice is independently associated with increased odds of mortality and organ dysfunctions.^9^ Here, we report on the novel independent association between *PCSK9* LOF genotype and endothelial dysfunction markers in the pediatric host with sepsis. Although several endothelial dysfunction markers were associated with LOF genotype, only Angpt-1 and VCAM-1 were independently associated after adjusting for age and complicated course. Furthermore, while the influence of *PCSK9* LOF genotype on VCAM-1 appears to be mediated through indirect effects on serum LDL and HDL, the association with Angpt-1 was independent of changes in serum lipoproteins concentrations. Finally, the effect of *PCSK9* LOF genotype on study mortality was not mediated by the canonical effect of patient genotype on LDL cholesterol but rather mediated by a non-canonical effect on Angpt-1.

Our data are strengthened by the observation that the influence of *PCSK9* LOF genotype on markers of endothelial dysfunction were more evident after excluding patients homozygous for an *LDLR* variant that renders it insensitive to PCSK9. Our data suggest the possible existence of an alternate role for the PCSK9-LDLR pathway that is critical to the host response in critical illness beyond hepatocyte-mediated bacterial and/or endotoxin clearance. We have previously demonstrated that juvenile *Pcsk9* null mice, challenged with sepsis, had a trend towards lower lipoprotein concentrations, higher bacterial burden in blood, and lower bacterial burden in the liver relative to the wildtype. Given the observational nature of this study, we were unable to ascertain whether the proclivity for greater endothelial dysfunction in the developing host with *PCSK9* LOF genotype is driven by a higher bacterial burden and related endothelial injury or a direct effect of PCSK9-LDLR pathway on the vascular endothelium.

The literature on the influence of PCSK9 inhibition on the vascular endothelium suggests both protective and potentially detrimental effects. Studies in macrovascular aortic endothelial cells (ECs) suggest that silencing PCSK9 may result in rescue of endothelial nitric oxide synthase (eNOS) production induced by lipopolysaccharide (LPS).^13^ Interestingly, the opposite was demonstrated in human umbilical vein endothelial cells (HUVECs).^22^ More recently Leung et al. demonstrated that PCSK9, in a dose-dependent manner through the LDLR, decreases the pro-inflammatory response to LPS in HUVECs.^14^ Our observational data demonstrate an association with Angpt-1, a key molecule involved in stabilizing endothelial barrier integrity.^23^ This finding warrants further study to elucidate the biological mechanisms at play. Taken together, the PCSK9-LDLR pathway, may have a potentially paradoxical response on vascular homeostasis, which may be high relevant to the host response among critically ill patients.

The lack of significant correlation of serum PCSK9 with endothelial dysfunction markers is consistent with our previous report where we noted only a weak association with the risk of complicated course in children with septic shock. Potential explanations for this discordance between patient genotype and serum protein concentrations with endothelial dysfunction markers include 1) although 90% of circulating PCSK9 is secreted by the liver, another major source of PCSK9 is vascular smooth muscle cells.^24^ Thus, it is conceivable that *PCSK9* LOF genotype results in lower local levels of PCSK9 essential to endothelial health, which are unmeasurable when sampling patient serum. 2) *PCSK9* LOF genotype may encode for different organ- and tissue- level receptor density of key downstream targets including LDLR. It is plausible that such variation may have a more significant effect on organ homeostasis and sepsis survival than serum PCSK9 concentrations during sepsis.

Our study has several limitations including 1) the observational nature of the study, 2) potential for linkage disequilibrium, 3) lack of assessment of dynamic changes in serum lipoproteins, PCSK9, and endothelial dysfunction markers, 4) potential for unadjusted confounders, and 5) fundamental biological differences with regard to lipoprotein metabolism such as the lack of cholesteryl ester transfer protein (CETP) among mice. Despite these limitations, our study highlights a novel association that warrants further study with due consideration of potential for host-developmental age and gene-environment interactions. First, increasing evidence in murine models suggest that downstream targets of PCSK9 including intra-cellular lipid transporters (LDLR) and vasculogenesis (Angpt-1) show a significant downregulation with increasing age.^25^ Accordingly, *PCSK9* LOF or pharmacological inhibition may have a considerably different effects according to the patient age. Second, adults may have a higher degree of circulating lipoproteins and comorbidities including dyslipidemia (oxidized HDL and LDL) at baseline. Accordingly, *PCSK9* LOF or pharmacological inhibition during sepsis may lead to the significant reduction in these dysfunctional lipids with consequent beneficial effects on endothelial dysfunction.^26^ On the contrary, further lowering of already low HDL and LDL among children, may result in drop below a critical threshold of these lipoproteins, which are essential for maintenance of vascular health and clearance of bacteria and endotoxin.

Recent results from a pilot trial testing the ‘Impact of PCSK9 Inhibition on Clinical Outcome in Patients During the Inflammatory Stage of the COVID-19’ (IMPACT-SIRIO 5; NCT04941105) demonstrate a survival benefit among adult patients.^27^ It is conceivable that such therapies will be trialed in other critically ill cohorts including sepsis and acute respiratory distress syndrome. Our genetic data indicate that PCSK9 inhibitors may not be biologically appropriate for use among critically ill children. Future mechanistic studies that investigate the PCSK9-LDLR-ANGPT-1 axis in the pediatric host may lead to the development of novel therapies aimed at restoring vascular homeostasis.

## Conclusions

We report on the independent association between *PCSK9* loss-of-function genotype and markers of endothelial dysfunction in a large cohort of critically ill children with septic shock with corroborative evidence in juvenile murine sepsis. After adjusting for the confounders, *PCSK9* LOF genotype was associated with lower Angpt-1 and higher VCAM-1 concentrations. After accounting for LDL and HDL concentrations, only Angpt-1 was significantly associated with *PCSK9* LOF genotype in pediatric septic shock, with evidence for causal mediation on sepsis mortality. Future mechanistic studies on the role of PCSK9-LDLR-ANGPT-1 pathway on vascular homeostasis may lead to the development of sepsis therapies specific to children.

## Figures and Tables

**Table 1.** Demographic and clinical characteristics of the cohort.

	LOF	Other	p value
n = 474, n (%)	195 (41.1%)	279 (58.9%)	
Age, median (IQR)	2.7 (0.8, 6.4)	2.6 (1.0, 5.9)	0.971
Sex, Male, n (%)	116 (56.9%)	159 (59.5%)	0.588
Race, Caucasian, n (%)	156 (80.0%)	196 (70.3%)	0.017
PRISM, median (IQR)	13 (8, 19)	11 (7, 18)	0.094
			
Any comorbidity, n (%)			
Malignancy, n (%)	21 (10.8%)	25 (9.0%)	0.513
Bone marrow transplant, n (%)	6 (3.1%)	10 (3.6%)	0.763
Steroids, n (%)	104 (53.3%0	139 (49.8%)	0.452
			
Complicated Course, n (%)	67 (34.4%)	68 (24.4%)	0.018
Mortality, n (%)	26 (13.3%)	21 (7.5%)	0.037
>2 Organ Failures, n (%)	76 (39.0%)	78 (28.0%)	0.012
PICU LOS, median (IQR)	7 (3, 14)	8 (3, 14)	0.496
PICU Free Days, median (IQR)	18 (5, 24)	19 (11, 24)	0.603

**Table 2.** Multivariate regression analyses to test the influence of PCSK9 LOF genotype on markers of endothelial dysfunction, accounting for age and complicated course as covariates. Patients homozygous for the rs688 LDLR variant were excluded.

Variable	N=	term	estimate	SE	P value
Angpt-1	403	Age	−0.132	0.287	0.647
	403	Complicated Course	−4.818	1.427	0.001
	403	LOF genotype	−4.515	1.824	0.014
					
Angpt-2	403	Age	−0.307	0.137	0.026
	403	Complicated Course	5.064	0.686	0.000
	403	LOF genotype	0.318	0.876	0.717
					
Tie-2	407	Age	−0.383	0.155	0.014
	407	Complicated Course	−1.752	0.772	0.024
	407	LOF genotype	−1.393	0.989	0.160
					
Angpt-2/Angpt-1	399	Age	−0.066	0.038	0.080
	399	Complicated Course	1.121	0.188	0.000
	399	LOF genotype	0.040	0.240	0.868
					
Angpt-2/Tie-2	403	Age	−0.009	0.012	0.470
	403	Complicated Course	0.404	0.062	0.000
	403	LOF genotype	0.074	0.079	0.345
					
sTM	407	Age	−0.236	0.078	0.003
	407	Complicated Course	2.962	0.389	0.000
	407	LOF genotype	0.878	0.498	0.078
					
ICAM-1	407	Age	−7.393	5.788	0.202
	407	Complicated Course	307.021	28.759	0.000
	407	LOF genotype	21.487	36.815	0.560
					
VCAM-1	405	Age	27.572	26.371	0.296
	405	Complicated Course	156.221	131.175	0.234
	405	LOF genotype	348.774	167.620	0.038

**Table 3.** Multivariate linear regression analyses to test the association between PCSK9 LOF genotype on endothelial dysfunction markers, accounting for age, complicated course, and serum LDL as covariates.

Variable	Term	Est.	SE	P value
Angpt-1	Age	−0.208	0.324	0.521
	Complicated Course	−3.612	1.679	0.032
	LDL	0.012	0.040	0.757
	LOF genotype	−5.429	2.083	0.010
				
Angpt-2	Age	−0.139	0.144	0.333
	Complicated Course	3.975	0.749	0.000
	LDL	−0.047	0.018	0.007
	LOF genotype	0.903	0.926	0.330
				
Tie-2	Age	−0.370	0.167	0.028
	Complicated Course	−0.600	0.868	0.489
	LDL	0.069	0.021	0.001
	LOF genotype	−1.135	1.078	0.293
				
Angpt-2/ Angpt-1	Age	−0.031	0.039	0.427
	Complicated Course	0.899	0.206	0.000
	LDL	−0.006	0.005	0.249
	LOF genotype	0.223	0.254	0.382
				
Angpt-2/ Tie-2	Age	−0.003	0.011	0.789
	Complicated Course	0.230	0.058	0.000
	LDL	−0.004	0.001	0.004
	LOF genotype	0.093	0.072	0.196
				
sTM	Age	−0.117	0.083	0.157
	Complicated Course	3.239	0.429	0.000
	LDL	−0.004	0.010	0.664
	LOF genotype	0.857	0.533	0.108
				
ICAM-1	Age	−1.860	5.818	0.749
	Complicated Course	298.604	30.164	0.000
	LDL	−0.164	0.713	0.819
	LOF genotype	55.212	37.479	0.142
				
VCAM-1	Age	47.267	28.422	0.097
	Complicated Course	62.357	147.532	0.673
	LDL	−7.597	3.472	0.029
	LOF genotype	340.612	182.877	0.063

**Table 4.** Results of Causal Mediation Analysis testing whether the effect of PCSK9 LOF genotype on 28-day mortality is mediated via Angpt-1, VCAM-1, or LDL.

Mediator	Direct effect of PCSK9 LOF (ADE)	p-value (ADE)	Effect due to Mediator (ACME)	p-value (ACME)	Total effect (ADE+ACME)	Mediator effect / Total effect
Angpt-1	0.074	0.012	0.010	0.0008	0.085	12.3 %
VCAM-1	0.081	0.009	0.005	0.170	0.085	5.5 %
LDL	0.086	0.004	<0.001	0.940	0.086	0.1 %

ADE: Average Direct Effects

ACME: Average Causal Mediation Effects (ACME)

Total effect: sum of ACME and ADE

## Data Availability

All data generated or analyzed during this study are included in this published article and its supplementary information files. The datasets used and/or analyzed during the current study are available are at through the Open Science Framework http://doi.org/10.17605/OSF.IO/TBDR9

## Abbreviations

PCSK9: Proprotein convertase subtilisin/kexin type 9
LDLR: Low density lipoprotein receptor
LOF: Loss-of-Function
GOF: Gain-of-function
LDL: Low Density lipoprotein
HDL: High Density Lipoprotein
Angpt-1: Angiopoietin-1
Angpt-2: Angiopoietin-2
Tie-2: TEK tyrosine kinase
ICAM-1: Intercellular Adhesion Molecule 1
VCAM-1: Vascular cell Adhesion Molecule
sTM: Soluble Thrombomodulin
